# NAFLD in normal weight individuals

**DOI:** 10.1186/s13098-022-00814-z

**Published:** 2022-03-24

**Authors:** Johanna K. DiStefano, Glenn S. Gerhard

**Affiliations:** 1 Diabetes and Fibrotic Disease Research Unit, Translational Genomics Research Institute, Phoenix, USA; 2 Lewis Katz School of Medicine, Temple University School of Medicine, Philadelphia, PA 19140 USA

**Keywords:** NAFLD, NASH, Lean, Nonobese, BMI, Prevalence, Clinical outcomes, Menopause, Choline deficiency, Genetic variation, Metabolic syndrome

## Abstract

Nonalcoholic fatty liver disease (NAFLD) can develop in lean individuals. Despite a better metabolic profile, the risk of disease progression to hepatic inflammation, fibrosis, and decompensated cirrhosis in the lean is similar to that in obesity-related NAFLD and lean individuals may experience more severe hepatic consequences and higher mortality relative to those with a higher body mass index (BMI). In the absence of early symptoms and abnormal laboratory findings, lean individuals are not likely to be screened for NAFLD or related comorbidities; however, given the progressive nature of the disease and the increased risk of morbidity and mortality, a clearer understanding of the natural history of NAFLD in lean individuals, as well as efforts to raise awareness of the potential health risks of NAFLD in lean individuals, are warranted. In this review, we summarize available data on NAFLD prevalence, clinical characteristics, outcomes, and mortality in lean individuals and discuss factors that may contribute to the development of NAFLD in this population, including links between dietary and genetic factors, menopausal status, and ethnicity. We also highlight the need for greater representation of lean individuals in NAFLD-related clinical trials, as well as more studies to better characterize lean NAFLD, develop improved screening algorithms, and determine specific treatment strategies based on underlying etiology.

## Introduction

Nonalcoholic fatty liver disease (NAFLD) is a chronic, progressive condition that arises from intrahepatic fat accumulation in the absence of monogenic metabolic disorders, infections, steatogenic medications, or significant alcohol consumption [1]. Since it was first described in 1980 [2], NAFLD has become the most common liver disease in Western populations [3, 4]. NAFLD is also recognized as a common chronic disease worldwide, affecting ~ 24% of the population [5], with a growing prevalence [6, 7]. In the United States, nonalcoholic steatohepatitis (NASH), a severe form of NAFLD characterized by hepatic inflammation and often accompanied by fibrosis, is the major cause of chronic liver disease and is emerging as the most common indication for liver transplantation [8].

Obesity is a risk factor for the development of NAFLD [9, 10] and the prevalence of NAFLD increases in parallel with BMI [11]. Nevertheless, NAFLD is not inextricably linked with obesity, as many individuals with obesity maintain normal intrahepatic content, while a significant number of lean individuals develop NAFLD, even in the absence of insulin resistance, type 2 diabetes (T2D), and related metabolic comorbidities [10]. Early observations by Bellentani et al. [12] noted a NAFLD prevalence of 16% in normal weight individuals enrolled in the Dionysos Study. One of the first studies in nonobese Asian populations reported a NAFLD prevalence > 23%, and many of the same characteristics observed in NAFLD patients with obesity, including male sex, higher BMI, older age, hyperuricemia, and elevated metabolic markers, were also common among nonobese patients [13–15]. Vos et al. [16] described the presence of NAFLD in nonobese individuals (BMI < 30 kg/m^2^) as a new clinical entity and defined it as “lean-NAFLD”. However, because body weight is not a component of the diagnostic criteria for NAFLD, and describing NAFLD itself as lean is imprecise, “NAFLD in lean individuals” has been suggested as a more accurate description of this condition [17]. In agreement, we utilize this terminology here.

Although NAFLD in lean individuals is not uncommon, the pathophysiology of the disease in lean patients remains poorly characterized. While some characteristics of NAFLD are shared among individuals in obese and lean BMI groups, not all lean individuals with NAFLD have metabolic antecedents that predispose to hepatic dysfunction. In these individuals, factors such as dietary composition, lifestyle factors, and genetic susceptibility may contribute to the development of NAFLD. Despite potentially distinct etiologies, NAFLD in lean individuals appears to follow a disease progression similar to that in patients with overweight or obesity, indicating that the absence of excess corporeal adiposity does not confer protection against hepatic inflammation, fibrosis, or decompensated cirrhosis. As discussed in the following sections, some studies have even reported more severe histological presentation and higher mortality in NAFLD patients with normal BMI compared to higher BMI groups.

Because NAFLD is a clinically silent disease in most cases, the absence of early signs and symptoms, coupled with normal laboratory and anthropometric measures, likely blind clinicians to the presence of NAFLD in lean individuals. However, lean NAFLD patients are at risk for progression to severe liver disease and possibly even increased mortality, warranting efforts to promote awareness of NAFLD in lean individuals. In this review, we summarize the literature on NAFLD prevalence, characteristics, outcomes, and mortality in lean individuals and discuss factors that may contribute to the development of NAFLD in this patient population. We also address clinical strategies for the screening and management of NAFLD in lean individuals based on suspected etiologies.

## Prevalence of NAFLD in lean individuals

The prevalence of NAFLD in lean individuals has been estimated almost exclusively using BMI as the sole criterion to describe body habitus. The international definition for normal BMI is defined as < 25 kg/m^2^. However, in Asians and Pacific Islanders, a BMI of < 23 kg/m^2^ is used due to the occurrence of visceral adiposity and risk of developing comorbidities, such as T2D and cardiovascular disease, at a lower BMI than that found in Europeans [18]. Using these thresholds to define lean individuals, a comprehensive survey of available studies based on BMI cut-offs of < 25 kg/m^2^ (< 23 kg/m^2^ for Asians) for lean individuals indicates that the prevalence of NAFLD ranges from 5 to 34% (Table 1). Of note, there is significant heterogeneity among these studies, which vary by geography, method of NAFLD determination, design, sample size, and comparison group. For example, the assignment of NAFLD was based on a number of different methods including liver biopsy, abdominal ultrasonography, computed tomography, liver function tests (i.e., hepatic transaminases), magnetic resonance imaging, controlled attenuation parameter, and several indices. Some studies were population-based, while others were hospital-based or located at a tertiary care clinic. Sample sizes ranged from 39 to more than 10,000, with the majority of studies emanating from South and East Asia. Comparator groups were either lean NAFLD compared to all lean or lean NAFLD compared to all NAFLD.

**Table 1:** Prevalence of NAFLD in individuals with normal BMI

Study	Country	Setting	Method	N	Comparison	Prev (%)
European/North American						
Bellentani et al. [12]	Italy	Population-based	US^a^	67	Lean NAFLD/all lean	16.4
Yououssi et al. [26]	USA	Population-based	US	4457	Lean NAFLD/all lean	7.4
Margariti et al. [29]	Greece	Tertiary clinic	US or biopsy	162	Lean NAFLD/all NAFLD	11.7
Chiloiro et al. [141]	Italy	Population-based	US	2946	Lean NAFLD/all lean	8.1
Cruz et al. [142]	USA	Hospital-based	Biopsy	1090	Lean NAFLD/all NAFLD	11.5
Denkmayr et al. [31]	Austria	Tertiary clinic	Biopsy	466	Lean NAFLD/all NAFLD	15.9
Hagström et al. [36]	Sweden	Hospital-based	Biopsy	646	Lean NAFLD/all NAFLD	19.0
Golabi et al. [37]	USA	Population-based	US or ION^b^	5375	Lean NAFLD/all lean i	10.8
Alferink et al. [143]	Netherlands	Population-based	US	3882	Lean NAFLD/all NAFLD	9.9
Zou et al. [38]	USA	Population-based	USFLI^c^	4711	Lean NAFLD/all NAFLD	4.9
Ahmed et al. [27]	USA	Hospital-based	Biopsy or imaging	4834	Lean NAFLD/all NAFLD	8.6
Younes et al. [33]	Multisite	Tertiary clinic	Biopsy	1339	Lean NAFLD/all NAFLD	14.1
Middle Eastern						
Akyuz et al. [144]	Turkey	Hospital-based	US or biopsy	483	Lean NAFLD/all NAFLD	7.6
Lankarani et al. [145]	Iran	Population-based	US	819	Lean NAFLD/all NAFLD	16.4
South Asian						
Singh et al. [15]	India	Tertiary clinic^h^	US	39	Lean NAFLD/all NAFLD	17.9
Das et al. [146]	India	Population-based	US and CT^d^	164	Lean NAFLD/all NAFLD	31.7
Kumar et al. [147]	India	Hospital-based	US	205	Lean NAFLD/all NAFLD	13.2
Bhat et al. [148]	India	Tertiary clinic	US and LFT^e^	150	Lean NAFLD/all NAFLD	15.3
Singh et al. [149]	India	Tertiary clinic	US	632	Lean NAFLD/all NAFLD	15.9
Alam et al. [150]	Bangladesh	Population-based	US	2782	Lean NAFLD/all NAFLD	14.5
Niriella et al. [108]	Sri Lanka	Population-based	US	936	Lean NAFLD/all NAFLD	12.8
Rahman et al. [151]	Bangladesh	Population-based	US	1305	Lean NAFLD/all NAFLD	4.1
Choudhary et al. [152]	India	Tertiary clinic^h^	Biopsy	157	Lean NAFLD/all lean	33.5
East Asian						
Kim et al. [14]	Korea	Population-based^h^	US	460	Lean NAFLD/all lean	16.1
Hsiao et al. [153]	Taiwan	Hospital-based	US	16,309	Lean NAFLD/all lean	32.1
Goh et al. [154]	Malaysia	Hospital-based^h^	US	1621	Lean NAFLD/all NAFLD	10.6
Feng et al. [155]	China	Hospital-based^h^	US	731	Lean NAFLD/all lean	18.3
Fukuda et al. [156]	Japan	Population-based^h^	US	4629	Lean NAFLD/all lean	4.6
Wang et al. [157]	China	Population-based	US	4899	Lean NAFLD/all lean	12.7
Yoshitaka et al. [158]	Japan	Hospital-based^h^	US	1647	Lean NAFLD/all NAFLD	22.1
Shao et al. [159]	China	Hospital-based	US and MRI	1509	Lean NAFLD/all NAFLD	20.2
Wang [160]	Japan	Hospital-based^h^	US	10,064	Lean NAFLD/ all lean	5.4
Meta-analysis						
Ye et al. [20]	Multinational	Multiple	US, CT, MRI^f^, CAP^g^, FLI^h^, HIS^i^, LPAI^j^, or biopsy	63,017	Lean NAFLD/all lean	10.6
Shi et al. [21]	Multinational	Population-based^h^	US	55,936	Lean NAFLD/all lean	10.2

Normal BMI: < 25 kg/m^2^ for non-Asians; < 23 kg/m^2^ for Asians

^a^US: abdominal ultrasonography

^b^ION: index of NASH

^c^USFLI: US fatty liver index

^d^CT: computed tomography

^e^LFT: liver function tests (i.e., hepatic transaminases)

^f^MRI: magnetic resonance imaging

^g^CAP: controlled attenuation parameter

^h^FLI: fatty liver index

^i^HIS: hepatic steatosis index

^j^LPAI: liver-spleen attenuation index

^h^Recruited from population of apparently healthy individuals or health checkup program

The highest NAFLD prevalence rates (e.g., > 30%) were observed in India, and were based on estimates obtained from community-based populations, indicating a lower degree of bias compared to studies in which participants were recruited from hospitals or tertiary liver clinics. This increased prevalence, coupled with observations that lean, healthy, sedentary, non-smoking Asian-Indian men have a three- to fourfold increased prevalence of insulin resistance associated with a twofold increase in hepatic fat content compared to Eastern Asian, Black, Caucasian, and Hispanic men [19], suggests that this lean population may be at particular risk for NAFLD due to yet-to-be-identified factors.

In addition to the individual studies in Table 1, a comprehensive meta-analysis using data from 84 studies (n = 10,530,308) found that within the NAFLD population, 19.2% (95% Confidence Interval [CI] 15.9–23.0) of participants were lean [20]. However, in the general population (23 studies; n = 113,394), comprising all individuals regardless of NAFLD status, only 5.1% (95% CI 3.7–7.0) had NAFLD in the presence of normal BMI. Among the lean population (19 studies; n = 49,503), 10.6% (95% CI 7.8–14.1) had NAFLD. The authors noted high heterogeneity among the results, although in general, European countries appeared to have the highest, and Asian countries the lowest, prevalence of NAFLD in nonobese individuals. In a similar analysis of 21 studies (N = 55,936), Shi et al. [21] estimated an overall NAFLD prevalence rate of 10.2% (95% CI 7.6–13.6%) in lean populations, also noting significant heterogeneity. Data from the Global NAFLD/NASH Registry comprising data from 18 countries found that approximately 8% of the patients were lean, based on BMI, and exhibited fewer components of the metabolic syndrome, fewer comorbidities, and less cirrhosis [22]. The year in which data were collected and sample size significantly impacted estimates of NAFLD prevalence, but BMI cut-off, region of study, population source, and method of diagnosis also exerted nonsignificant effects on the observed heterogeneity. Like obesity-associated NAFLD [6], the prevalence of NAFLD in lean individuals is also increasing. In studies with data collected before 2000, the prevalence was 5.6%, but from 2001 to 2010, and after 2011, rates were estimated at ~ 11.0% and 12.6%, respectively, reflecting the trend of increasing prevalence in the overall population [23].

Several groups have estimated NAFLD prevalence in nonobese populations using combined normal and overweight BMI groups (i.e., < 30 kg/m^2^ [< 25 kg/m^2^ in Asians]). In China, a population study comprising 911 individuals recruited from the census database of the Hong Kong government reported a NAFLD prevalence of 19.3% in nonobese subjects [24]. In a cross-sectional study of individuals receiving health checkups in Japan, NAFLD prevalence in nonobese individuals was estimated at 15.2% [25]. NAFLD prevalence in nonobese, nondiabetic Belgians undergoing biopsy for chronic liver disease was 2.8% (50/1777) [16]. Estimates in nonobese populations are similar to those found in lean populations (Table 1), revealing potential limitations of BMI cut-off for NAFLD screening in the general population.

## Clinical characteristics, outcomes, and mortality of lean individuals with NAFLD

### Clinical characteristics

A number of studies have compared clinical characteristics between lean and non-lean NAFLD cohorts. An early study by Vos et al. [16] observed the presence of NASH and fibrosis in 61% and 55% of the lean group, respectively. Relative to healthy controls, these individuals were less insulin sensitive and had higher triglyceride levels. However, these observations were based on a BMI cut-off < 30 kg/m^2^ for lean individuals and a relatively small sample size (31 “lean” and 48 obese individuals with NAFLD and eight healthy controls), thereby, limiting the conclusions to be drawn.

A survey of available studies indicates that, in general, lean individuals with NAFLD have a more favorable metabolic profile compared to those with a higher BMI (Table 2). Measures of metabolic markers such as waist circumference, triglyceride levels, fasting plasma glucose, HDL-C (high-density lipoprotein-cholesterol), adiponectin levels, and Homeostatic Model Assessment for Insulin Resistance (HOMA-IR) appear to be intermediate between lean individuals without NAFLD and those with both NAFLD and obesity (Table 2). Many studies report a male dominance and younger age relative to the non-lean group. Multivariate analysis of data from the National Health and Nutrition Examination Survey III (NHANES III) showed that lean individuals with NAFLD were more commonly Hispanic with T2D and hypertension compared to lean control individuals without liver disease, but relative to individuals with overweight or obesity, NAFLD in lean individuals was independently associated with younger age, female sex, and a lower prevalence of insulin resistance and hypercholesterolemia [26]. Among NAFLD patients living in Olmstead County (Minnesota), female predominance (66.9%) and a higher proportion of Asian and African American individuals (13.2%) were found in lean individuals compared to overweight (47.2%; 6.5%) and obese (56.1%; 7.4%) groups [27]. Feldman et al. [28] reported lower serum levels of lysophosphatidylcholines and phosphatidylcholines and higher levels of glutamate in lean individuals with NAFLD relative to lean healthy controls, but no differences in level of physical activity or frequency of fast-food consumption were observed between the two weight groups. In this study, lean individuals with NAFLD had levels of severely impaired glucose tolerance almost identical to those in found in NAFLD patients with obesity, and ~ 30% were diagnosed with T2D. Glucose homeostasis thus appears to be abnormal in some lean subjects with NAFLD, confirming data suggesting that the accumulation of liver fat may be of particular importance to the development of insulin resistance and diabetes even in the absence of obesity [28].

**Table 2:** Characteristics and mortality associated with NAFLD in lean individuals

Study	Country	Age^a^	BMI	ALT (U/L)^a^	AST (U/L)^a^	Metabolic profile and related characteristics^b^	Mortality	F/U^c^
European/North American								
Younossi et al. [26]	USA	41.9 ± 1.2	22.2 ± 0.16	18.0 ± 0.16	21.5 ± 0.16	Younger, female predominance, lower ALT, AST, HOMA score, platelet count, lower frequency of visceral obesity, insulin resistance, T2D, hypercholesteremia, and hypertension	–	–
Margariti et al. [29]	Greece	44 ± 16	NA	92 (17–164)	45 (18–121)	Higher ALT and AST, less comorbidities, smaller waist circumference, lower liver stiffness measures	–	–
Cruz et al. [142]	USA	NA	23.1 ± 1.7	NA	NA	Male predominance, non-Caucasian, lower ALT levels and HOMA, lower prevalence of T2D, hypertension, hypertriglyceridemia, low-HDL-C, central obesity, and metabolic syndrome, lower degree of steatosis and less advanced fibrosis, more severe lobular inflammation	Higher overall mortality than non-lean NAFLD patients	11.1 ± 6.8
Feldman et al. [28]	Austria	61 (12)^d^	23.6 (1.8)^d^	21.0 (14.0)^d^	22.0 (10.5)^d^	Waist circumference, ALT, GGT, TG, HDL-C, FPG, adiponectin levels, and HOMA-IR intermediate between lean healthy and NAFLD with obesity	–	–
Fracanzani et al. [30]	Italy	46 ± 13	23 ± 2	64 ± 42	41 ± 27	Less prevalence of hypertension, diabetes, and metabolic syndrome, NASH, fibrosis of F2 or higher, carotid plaques and significantly thinner carotid intima-media	–	–
Denkmayr et al. [31]	Austria	48.7 ± 14.8	23.1 ± 1.5	60.0 ± 36.4	43.0 ± 26.9	Less components of metabolic syndrome, higher rate of cirrhosis	–	–
Bernhardt et al. [161]	Germany	NA	23.8 (23.0–24.7)	26.0 ± 7.1	26.4 ± 3.0	Higher serum ferritin, hemoglobin, hematocrit, and mean corpuscular hemoglobin concentration, lower levels of soluble transferrin receptor, high HOMA-IR, TC, LDL-C, and TG, comparable to NAFLD patients with obesity	–	–
Hagstrom et al. [36]	Sweden	51.4 ± 13.4	23.1 ± 2.7	72 ± 47	44 ± 25	Older, lower transaminase levels, lower stages of fibrosis, and lower prevalence of NASH at baseline; increased risk for severe liver disease	Similar overall mortality as non-lean NAFLD patients	19.9
Golabi et al. [37]	USA	50.9 ± 1.3	NA	NA	NA	Older, more likely to be Hispanic, had lower income, and had reported poorer health and more comorbidities^e^	Higher risk for all cause and CV mortality compared to lean controls	17.8
Feldman et al. [162]	Austria	47.6 ± 14.5	23.0 ± 1.5	54.6 ± 35.5	47.6 ± 24.8	Higher likelihood of dying from liver-related causes compared NAFLD patients with overweight or obesity; higher proportion with cirrhosis; less features of metabolic syndrome	Similar overall mortality as NAFLD patients with obesity	8.4
Ahmed et al. [27]	USA	51.5 ± 18.0	22.5 ± 2.0	NA	NA	Female predominance, higher proportion of Asian and African Americans, lower risk of metabolic comorbidities; same risk of cirrhosis and decompensation, malignancy, and cardiovascular events as non-lean NAFLD patients	Higher risk for all-cause mortality compared to NAFLD patients with obesity	6.4
Younes et al. [33]	USA	45 (36, 55)^d^	29.8 (26.5, 34.5)^d^	59 (41, 88)^d^	38 (28, 54)^d^	Younger, male predominance, less steatosis, lobular inflammation, ballooning and advanced liver fibrosis	Same mortality risk as non-lean NAFLD patients	7.7
Middle Eastern								
Akyuz et al. [144]	Turkey	41.2 ± 11.8	23.6 ± 1.3	82 ± 46	49 ± 38	Younger, lower blood pressure, higher hemoglobin, lower prevalence of metabolic syndrome, and less severe hepatic fibrosis	–	–
Lankarani et al. [145]	Iran	49.8 ± 13.9	NA	NA	NA	Lower waist circumference, TG levels, and prevalence of metabolic syndrome	–	–
South Asian								
Kumar et al. [147]	India	38 ± 15.4	21.3 ± 1.9	45 (11–217)	38 (23–180)	Similar metabolic profile as overweight group, but lower than obese group, lower prevalence of fibrosis in lean individuals, but similar prevalence of NASH	–	–
Bhat et al. [148]	India	39.9 ± 7.4	21.7 ± 1.3	69.9 ± 29.8	NA	Lower HOMA-IR, similar levels of fasting and 2 h glucose and lipid levels	–	–
Niriella et al. [108]	Sri Lanka	35.6 ± 6.4	NA	NA	NA	Male predominance, lower prevalence of hypertension and central obesity, similar prevalence of other metabolic comorbidities as non-lean group	–	–
Choudhary et al. [152]	India	33.5 ± 10.4	21.3 ± 1.2	33.4 ± 11.7	26.6 ± 7.5	Younger age, lower ALT, TGs, fasting glucose, LDL-C, higher HDL-C than non-lean group	–	–
East Asian								
Kim et al. [14]	Korea	51.6 ± 9.7	23.4 ± 1.3	31.9 ± 19.0	23.5 ± 7.7	Male predominance, higher BMI, WC, WHR, uric acid, fasting blood glucose, insulin, ASP, ALT, HOMA-IR compared to lean controls; no differences in metabolic variables compared to overweight group	–	–
Feng et al. [155]	China	48.2 ± 10.5	22.7 ± 1.1	21.6 ± 11.9	21.1 ± 9.3	Male predominance, higher BMI and blood pressure, and greater likelihood of having diabetes, metabolic syndrome, and hypertension compared to lean controls; lower levels of blood glucose, blood pressure, hyperlipidemia, IR, blood cell count and HGB than non-lean NAFLD patients	–	–
Fukuda [156]	Japan	42.6 ± 7.6	21.8 ± 0.9	25 (19, 36)	19 (15, 23)	Male predominance, higher incidence of T2D, ALT, AST, HbA1c, FPG, and TG compared to overweight group without NAFLD and lean control group	–	12.8
Wang et al. [157]	China	43 (32–58)	21.6 (20.2–22.8)	16.1 ± 12.3	20.3 ± 8.0	Male predominance	–	–
Yoshitaka et al. [158]	Japan	50.0 ± 7.9	22.0 ± 0.7	30.2 ± 15.9	21.1 ± 6.1	Higher blood pressure, triglycerides, fasting plasma glucose, uric acid, ALT, AST, and GGT, lower HDL-C, higher risk of incident CVD relative to lean controls	–	–
Shao et al. [159]	China	44.7 ± 11.9	21.6 ± 1.2	24 (19, 35)	25 (20, 32)	Lower WC, WHR, blood pressure, ALT, AST, fasting insulin, HOMA-IR, atherosclerosis index, liver fat content, and liver stiffness compared to nonlean group with NAFLD		
Wang [160]	Japan	45.6 ± 8.3	21.7 ± 1.1	16.2 ± 11.5	17.2 ± 8.4	Male predominance, older age, higher BMI, WC, smoking status, FPG, HbA1c, TG, blood pressure, hepatic transaminases, and risk of incident T2D, and lower HDL-C compared to lean control group		
Meta-analyses								
Sookoian and Pirola [34]	Multi	NA	NA	NA	NA	Lower fibrosis score, less risk for NASH, and lower NAFLD activity compared to NAFLD patients with obesity	–	–

BMI: < 25 kg/m^2^ for non-Asians; < 23 kg/m^2^ for Asians

*NA* information not available

^a^Mean (standard deviation) unless indicated otherwise

^b^Compared to NAFLD patients with obesity, unless noted otherwise

^c^Average follow-up in years

^d^Median (interquartile rang [IQR])

^e^Compared to lean non-NAFLD controls

Despite the better metabolic profile generally observed in lean individuals with NAFLD relative to those with obesity, the risk of disease progression to NASH is comparable to that experienced in NAFLD patients with overweight or obesity. In 56 Greek subjects with liver biopsy-documented NAFLD, the severity of inflammation and fibrosis did not differ between weight groups and NASH prevalence in lean individuals was 50% (compared to 68.8% in the non-lean NAFLD group) [29]. Another study found that 42% of lean Italian NAFLD patients had NASH, of which 42.3% had a fibrosis score of 2 or higher [30]. In lean Austrians with NAFLD, rates of portal inflammation, lobular inflammation, hepatocyte ballooning, perisinusoidal and periportal fibrosis, and NASH were similar between lean and non-lean groups [31, 32], although the proportion of lean individuals with cirrhosis was significantly higher compared to non-lean groups (11% vs. 2–3%) [32]. In NHANES III participants, individuals with normal BMI had the same risk of cirrhosis and decompensation, malignancy, and cardiovascular events as those in the overweight and obese categories, indicating that in these individuals, a normal BMI does not confer protection against progression or severity of liver dysfunction in the context of NAFLD [27].

In contrast, a multinational study found that lean subjects with NAFLD had significantly less steatosis, lobular inflammation, ballooning, and advanced liver fibrosis compared to the non-lean group, although 50% and 10% of lean individuals displayed mild/moderate fibrosis and advanced fibrosis, respectively [33]. Findings from meta-analyses studies were in agreement with a more favorable metabolic profile and milder disease progression in lean individuals with NAFLD. Sookoian and Pirola [34] reported that lean NAFLD patients showed less severe histological features than NAFLD patients with overweight or obesity and were less likely to have NASH. However, 33% of lean individuals with NAFLD had NASH, and it should be noted that in this analysis, all but one of the studies in Asian populations failed to use the BMI cut-off point of < 23 kg/m^2^, and therefore, included overweight individuals in the estimates. Shi et al. [21] found that lean and nonobese NAFLD patients were predominantly male and had a significantly lower rate of hypertension, lower waist circumference, lower levels of fasting plasma glucose, triglycerides, and uric acid, and higher levels of HDL-C compared to NAFLD patients with obesity. No significant differences were observed between these two groups with respect to diabetes prevalence, age, and levels of total cholesterol and low-density lipoprotein-cholesterol (LDL-C), suggesting that lean/nonobese NAFLD patients may have a risk for developing metabolic diseases similar to NAFLD patients with obesity. Alam et al. observed that lean and non-lean NAFLD patients had similar characteristics and shared common risk factors [35].

### Outcomes and mortality

Few studies have investigated differences in outcomes and mortality between lean and non-lean individuals with NAFLD (Table 2). Lean Swedish patients with NAFLD, despite a better prognostic profile at baseline, including a lower prevalence of NASH and advanced fibrosis, showed an increased risk for development of severe liver disease during follow-up compared to patients with a higher BMI, even after adjustment for age and fibrosis stage at baseline [36]. This unexpected finding may indicate that the lean individuals in this study experienced a faster rate of fibrosis progression relative to patients with a higher BMI. In a cohort of 1339 NAFLD patients from Australia, Italy, Spain, and the United Kingdom, followed for a median period of 7.6 years, new onset diabetes, cardiovascular events, extrahepatic cancers, liver-related events, and hepatocellular carcinoma (HCC) occurred in 6.2%, 7.3%, 4.7%, 8.9%, and 1.0% of lean individuals, respectively [33]. The incidence of these complications, as well as overall survival, were not significantly different between normal and high BMI groups.

Using data from the NHANES III with a median follow-up period of 17.8 years, Golabi et al. [37] reported that the weighted, unadjusted all-cause mortality was significantly higher in lean individuals with NAFLD compared to lean individuals without NAFLD (40.9% vs. 17.9%, *P* < 0.001). In lean NAFLD patients, the unadjusted hazard ratio (HR) for all-cause mortality was 2.44 (95% CI 1.77–3.37), which remained significant after adjusting for demographic variables, metabolic components, and primary comorbidities. Likewise, weighted unadjusted cardiovascular mortality was also significantly higher in lean individuals with NAFLD (15.1% vs. 3.7%, *P* < 0.001), showing a 238% increased risk of cardiovascular mortality, following adjustment. In 299 Austrian NAFLD patients (38 lean, 165 overweight, and 93 obese) over a follow-up period of 8.4 years, lean individuals had a lower overall mortality compared to overweight patients, but a mortality rate similar to NAFLD patients with obesity [32]. Notably, in this population, lean patients had a significantly higher mortality rate from liver-related causes compared to the overweight (11% vs. 4%) and obese groups (11% vs. 4%). In NAFLD patients from Olmstead County, the normal BMI group had a higher risk of death relative to the high BMI group [27]. The most common causes of death in the normal BMI group were malignancy (25.7%), cardiovascular event (21.6%), and infection (13.5%). In contrast to the findings reported by Feldman et al. [32], who reported significantly higher numbers of fatal liver-related events in the lean BMI group compared to higher BMI groups, mortality due to hepatic events was significantly lower in the normal BMI group (1.4%) compared to the obese BMI group (10.4%), but similar to the overweight BMI group (2.0%), and no significant differences in mortality from cardiovascular events or malignancy were observed among the three groups. A recent study by Zou et al. [38] found that lean NAFLD patients had the highest 15-year cumulative all-cause mortality (76.3%) compared to nonobese NAFLD patients (51.7%), NAFLD patients with obesity (27.2%), and individuals without NAFLD (20.7%). The analysis revealed similar patterns related to cardiovascular disease (16.9% vs. 5.6% vs. 8.8%, respectively, P = 0.0013).

Combined, these studies indicate that despite lower adiposity, less severe dyslipidemia, and lower levels of hepatic transaminases, lean individuals with NAFLD are at similar or greater risk as those with higher BMI for cardiovascular disease, malignancy, progressive liver disease, and increased all-cause mortality associated with NAFLD. The reason(s) for this relative increase in risk has not yet been characterized and may likely depend on the underlying pathogenesis of NAFLD in lean individuals.

## Possible causes of NAFLD in lean individuals

**Fig. 1:**
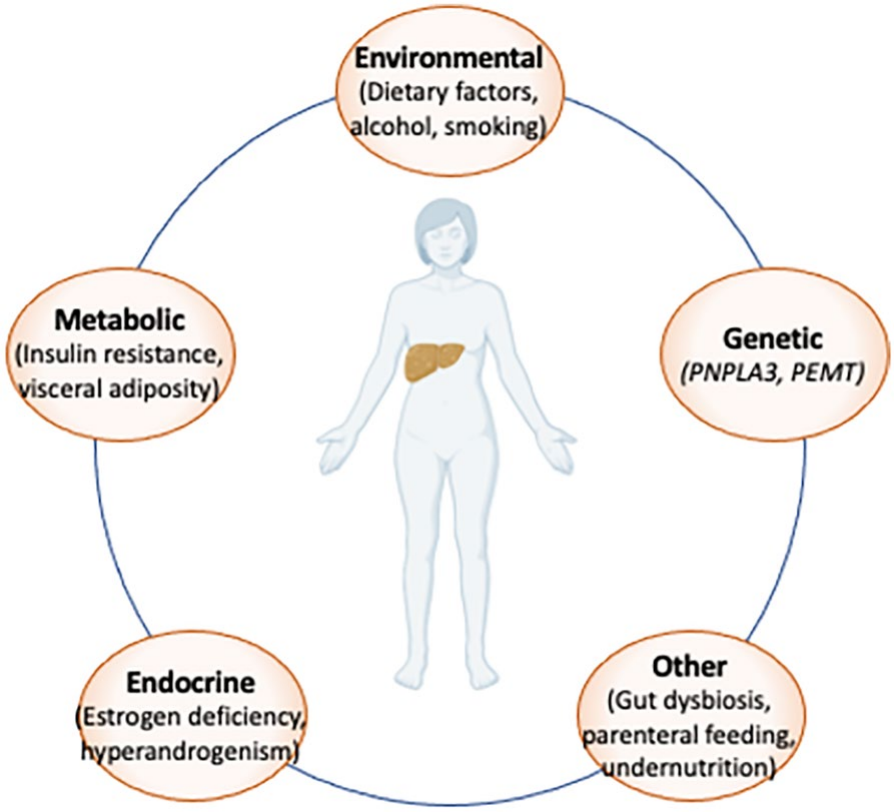
Potential contributors to NAFLD in lean individuals. Despite the same pathological findings in the liver, the factors that contribute to NAFLD and subsequent progression NASH in lean individuals are not yet well-characterized relative to those with obesity-related NAFLD. However, a number of factors that likely influence risk of NAFLD development and progression, even in the absence of excess adiposity, have been postulated in the recent literature. The majority of these risk factors fall into environmental, endocrine, genetic, and metabolic origins. Most of these factors are also expected to interact with one another, as well as other, as-yet-undefined factors, to further modulate NAFLD risk

Recent epidemiological and clinical studies have identified a number of factors that may contribute to the development of NAFLD in the absence of excess adiposity (Fig. 1). The main classes of these factors include environmental determinants, of which the role of dietary composition has been the best studied, genetic susceptibility, endocrine dysfunction, and metabolic derangement. Some of these factors are known to interact with one another to modulate NAFLD risk, oftentimes in the presence of increasing visceral adiposity, regardless of BMI, suggesting a common metabolic pathway that underlies NAFLD development in all individuals regardless of body habitus.

### Environmental factors

Excessive intakes of sucrose, refined carbohydrates, saturated fats, and animal protein are generally regarded as major factors in the development of NAFLD [39–44]. In particular, regular consumption of sugar-sweetened beverages is strongly associated with NAFLD in adults [39, 45–47] and children [48]. In adults, soft drink consumption predicts NAFLD, even following adjustment for dietary composition and physical activity level [47]. Soft drink consumption in NAFLD patients with no risk factors for metabolic syndrome was three times higher than in healthy controls and was significantly associated with the presence of fatty liver [49].

Fructose is a monosaccharide that, together with glucose, forms sucrose (table sugar). Regular dietary fructose consumption induces hepatic de novo lipogenesis and endoplasmic stress, impairs fatty acid oxidation, depletes beneficial microbial species in the gut [50], and promotes hepatic inflammation through the generation of both uric acid and gut-derived endotoxins [51–55]*.* Due to the constellation of these metabolic effects, dietary fructose may indirectly promulgate hepatic insulin resistance, an important factor in the development of NAFLD [52]. The relationship between dietary fructose and NAFLD is an area of active investigation [56–63]. For example, in adults with NAFLD, daily fructose consumption was associated with higher fibrosis stage [64], while in children, high fructose intake was independently associated with NASH [65].

Thus far, dietary interventions for NAFLD have been limited to non-lean individuals. In a comparison of fat- and carbohydrate-restricted diets in adults with obesity and NAFLD, only the latter led to significant reductions in hepatic fat fraction [66]. Interestingly, the carbohydrate-restricted group also exhibited significantly greater reductions in insulin resistance, abdominal fat mass, and body fat mass compared to the fat-restricted group [66]. Eight weeks of sugar restriction in adolescent males with NAFLD yielded significant decreases in hepatic fat fraction, mean body weight, and mean levels of alanine aminotransferase (ALT), aspartate aminotransferase (AST), gamma-glutamyl transferase (GGT), and total cholesterol [67]. Reduced liver fat was independent of changes in weight or measures of adiposity [67]. Similarly, isocaloric substitution of starch for sugar over a period of 9 days resulted in reduced levels of liver fat, visceral adipose tissue, fractional de novo lipogenesis, and insulin resistance in children with obesity who reported habitually high (> 50 g/day) sugar consumption [68]. The strongest effects of a low fructose, low glycemic index, and low glycemic load diet on metabolic parameters were observed in children with NASH, who also had the highest reported dietary intakes of fructose at baseline, compared to children with NAFLD, and healthy controls [69]. Consistent with these results, current EASL guidelines now recommend a Mediterranean diet with avoidance of processed foods and added fructose for individuals with NAFLD [3].

Some dietary components have been associated with NAFLD in lean individuals. Deprivation of choline, an essential nutrient for human health [70], for 3 weeks in healthy male volunteers resulted in increased ALT activity [71]. Extended depletion of choline for up to 42 days resulted in significant liver dysfunction in men and postmenopausal women [72–74]. Postmenopausal women from the NASH Clinical Research Network study with low dietary choline intake had worse fibrosis (OR 3.37; p = 0.002) after adjusting for age, race, obesity, triglycerides, alcohol, and steroid use [75]. In normal-weight Chinese women, higher dietary choline intake was associated with a lower risk of NAFLD [76]. The Adequate Intake (AI) for choline is 550 mg/day for men and 425 mg/day for women, although analyses of dietary patterns reveal that the vast majority of individuals do not achieve these levels [76–78]. Because choline is present at more abundant levels in animal-derived foods, vegetarians and vegans may have a greater risk of deficiency, and therefore a higher risk of NAFLD due to inadequate intake [77, 78].

In the choline biosynthesis pathway, phosphatidylethanolamine *N*-methyltransferase (PEMT) catalyzes the formation of phosphatidylcholine, which is required for the secretion of VLDL (very low-density lipoprotein-cholesterol) in the liver [79]. *PEMT*^*−/−*^ mice fed a diet high in fat and sucrose rapidly develop hepatic steatosis, inflammation, and fibrosis; dietary choline supplementation ameliorated liver damage in these animals [80–84]. In humans, hepatic *PEMT* expression was observed to be lower in NASH patients compared to NAFLD patients, and was significantly correlated with platelet counts, which decline in tandem with progression of fibrosis [80]. Lower hepatic *PEMT* expression was also associated with lower BMI [80], in keeping with the demonstration that *PEMT*^*−/−*^ mice are protected from high fat diet-induced obesity and insulin resistance [84, 85]. Because the *PEMT* gene is regulated by estrogen [86, 87], the detrimental impact of choline deficiency may be exacerbated following the menopausal transition, consistent with findings from choline depletion [73, 88] and observational studies [75]. Together, findings from human and mouse studies suggest that choline deficiency, whether due to low *PEMT* expression or inadequate dietary intake, may be related to the development of NAFLD and a susceptibility toward progressive disease specifically in lean individuals.

Other environmental factors that may modulate NAFLD include alcohol consumption and cigarette smoking. The diagnostic criteria for NAFLD requires the absence of significant alcohol consumption, although there is not yet a global consensus among professional societies on what level is considered “significant”. In the United States, alcohol limits for men and women are 294 g/week and 196 g/week, respectively [89], while in Europe and Asia, limits are 210 g/week (men)/140 g/week (women) [3] and 140 g/week (men)/70 g/week (women) [3, 90, 91], respectively. However, a retrospective study using prospectively recorded data found that French NAFLD patients who consumed more than 7 units/week (i.e., 56 g) experienced a significantly higher mortality than those consuming less, and conversely, alcohol consumption of less than 1 unit/week (8 g) was significantly associated with improved survival in NAFLD patients [92]. In the NAFLD population, alcohol consumption in excess of 7 units of alcohol/week significantly increased the probability of death from cardiovascular causes and was associated with a significantly higher rate of overall complications, as well as cardiovascular- and cancer-related complications [92]. Moderate alcohol consumption (30 g/day for men and 20 g/day for women) has also been associated with worse fibrosis in NAFLD [93]. Other large and multiethnic studies have likewise reported a significant relationship between low-to-moderate alcohol consumption and increased mortality and morbidity risk [94–96]. Interestingly, alcohol consumption worsens liver disease in individuals with obesity. A BMI > 30 kg/m^2^ was found to double the hepatoxicity of alcohol [97] and synergistically increase the risk of HCC [98]. The role of modest alcohol consumption and NAFLD in lean individuals has not yet been investigated.

Cigarette smoking is associated with NAFLD onset [99], progression to fibrosis, and increased risk for severe liver disease [100–102]. In Chinese men with a history of heavy smoking and moderate alcohol consumption, NAFLD risk was 85% higher compared to individuals who neither drank nor smoke [103].

### Genetic susceptibility

Obesity is the strongest independent risk factor for NAFLD, even after adjusting for age, sex, total cholesterol, triglycerides, HDL, LDL, glucose, uric acid, homocysteine, creatinine, AST, ALT, and hypertension [11]. However, even in the presence of severe obesity and the corresponding chronic caloric excess, some individuals do not develop NAFLD. Conversely, the relationship between risk of NAFLD and BMI is J-shaped, with risk increasing below BMI of 19 from a nadir [11]. These data suggest that there are putative NAFLD susceptibility and/or protective factors that can modify the effects of BMI, particularly genetic variation [104]. The single nucleotide variant (SNV) resulting in the I148M substitution (rs738409) in the patatin-like phospholipase domain-containing protein 3 gene (*PNPLA3*) is the major genetic risk factor for NAFLD known to date [105]. In addition to *PNPLA3*, variants in *MBOAT7 (*membrane- bound *O*-acyltransferase domain-containing 7) and *TM6SF2* (trans-membrane 6 superfamily antigen 2) have been associated with NAFLD [106]. Most genetic association studies have been performed in individuals with classical obesity-related NAFLD and few data are available from lean populations.

In a study of 904 community dwelling Japanese participants, in whom the prevalence of NAFLD was 12.4%, 41.4% and 59.1% in lean, overweight, and obese groups, the *PNPLA3* rs738409 risk genotype (GG) increased NAFLD risk in lean subjects by more than twofold compared with overweight and obese participants [107]. No differences in risk were found for the *MBOAT7* or *TM6SF2* NAFLD risk alleles after stratifying by BMI. In a study of 187 Austrian participants, Feldman et al. [28] observed a higher rate of *PNPLA3* risk alleles in lean individuals with NAFLD compared with the lean control group, with a frequency comparable to NAFLD patients with obesity. In addition, a significantly greater proportion of lean individuals with NAFLD carried the rs58542926 risk allele (4%), relative to non-lean NAFLD patients (0.3%). In Italian patients with lean NAFLD, the only variable associated independently with NASH and a fibrosis score ≥ 2 was the presence of the rs738409 (*PNPLA3*) risk allele [30]. The rs738409 risk genotype was also associated with NAFLD in lean individuals in a Sri Lankan population [108] and nonobese Japanese subjects [109]. A recent study found the highest NAFLD risk increase among carriers of the rs738409 risk genotype in 529 lean subjects (OR 6.04, 95% CI [2.62, 13.91]), compared with 162 and 213 individuals with overweight (OR 3.43, 95% CI [1.06, 11.14]) and obesity (OR 2.51, 95% CI [0.93, 6.78]), respectively [110]. Other groups have not found evidence for statistically significant differences for NAFLD risk alleles in *PNPLA3* and *TM6SF2* between weight groups [31, 111].

A risk genotype (AA) at the V175M variant (rs7946) in *PEMT*, which results in a partial loss of PEMT activity, was 1.7-fold higher in individuals with NAFLD compared to normal controls [112]. Additional *PEMT* variants (rs12325817, rs4646343, and rs3761088) were associated with developing liver dysfunction in response to a choline-depleted diet [88]. Two other *PEMT* variants, rs1531100 and rs4646365, increased liver damage risk in postmenopausal women [88]. Variants in genes from the choline biosynthesis pathway, namely choline kinase A, moderated the effects of a low choline diet [88], while a genetic signature comprised of variants in the choline and 1-carbon metabolism pathways were associated with severity of hepatic steatosis [113]. Using an unbiased exome sequencing approach in a discovery set of two lean NAFLD patients and six lean controls, only rs7946 (*PEMT*) and rs2290532 in oxysterol-binding protein-related protein 10 (*OSBPL10*) were found to be associated with NAFLD [114]. Genotyping in a validation cohort of 191 lean individuals with NAFLD and 105 lean controls revealed a threefold higher risk of NAFLD in carriers of the rs7946 risk genotype, but no significant differences were found for the *OSBPL10* variant.

Lipodystrophies are a group of heterogeneous rare genetic disorders characterized by the common phenotype of deficient adipose tissue without nutritional deprivation or increased metabolism [115]. The inability to store lipids as fat leads to several adverse complications including NAFLD and liver fibrosis, which can lead to cirrhosis. Pathogenic variants in several genes can cause familial partial lipodystrophies including peroxisome proliferator-activated receptor gamma (*PPARG*), lamin A/C (*LMNA*), perilipin 1 (*PLIN1*), hormone-sensitive lipase (*LIPE*), cell death-inducing DFFA-like effector C (*CIDEC*), and Akt murine thymoma viral oncogene homolog 2 (*AKT2*) [116]. Hepatic steatosis is an almost universal finding in these individuals, suggesting that NAFLD in lean individuals may be a type of ectopic fat deposition similar to lipodystrophy. Genetic evidence supports such a mechanism. A polygenic risk score associated with insulin resistance and decreased adiposity in the lower extremities, both of which are features of lipodystrophy, has been reported [117]. Subsequently, Chen et al. [118] determined that the lipodystrophy polygenic risk score was associated with NAFLD, increased liver fibrosis, and decreased lower extremity fat mass.

### Other factors

NAFLD can develop against the backdrop of endocrine disturbances, often by exacerbating hormone-related metabolic alterations. For example, women are at high risk of developing NAFLD and NASH following the menopausal transition [119], likely due to the loss of protection conferred by estrogens, in combination with sub-clinical disturbances in metabolic parameters prior to menopause, such as mild to moderate levels of adiposity, dyslipidemia, and impaired glucose tolerance. Hyperandrogenism is also associated with hepatic steatosis and fibrosis in women, independent of insulin resistance and adiposity [120, 121], although increased circulating testosterone levels in middle-aged women are associated with higher visceral adiposity [122]. Hyperthyroidism-induced NAFLD is regarded as a distinct disease entity [123] and thyroid hormone supplementation improves liver dysfunction [124]*.* To date, no studies have specifically focused on the role of endocrine factors in NAFLD risk in lean individuals, although many changes in hormonal levels are accompanied by increasing visceral adiposity, which yields ramifications for NAFLD susceptibility, even in lean individuals.

There is some evidence suggesting that high dietary fat or fructose intake in animals can synergistically enhance the effects of estrogen deficiency, leading to exaggerated effects on hepatocellular injury [125, 126]. Similarly, interactions between choline deficiency and hormonal status may modulate NAFLD risk. For example, postmenopausal women had significantly worse fibrosis compared to premenopausal women, although both groups had similarly low levels of choline intake [75]. As noted above, reduced endogenous production of estrogen results in diminished *PEMT* expression, which may lead a greater susceptibility to the development of NAFLD in postmenopausal women with chronic states of choline deficiency [86, 87].

Other potential etiologies of NAFLD in lean individuals include those related to the gut dysbiosis, parenteral nutrition, undernutrition, and specific steatogenic medications. These have been discussed in detail elsewhere [127–131].

## Screening and clinical management of NAFLD in lean individuals: outstanding questions

Despite the prevalence and adverse outcomes associated with NAFLD in normal weight individuals, there are no global consensus guidelines for NAFLD screening, nor is screening in the general population recommended by any professional societies. As shown in Table 3, practice guidance statements developed by the American Association for the Study of Liver Diseases (AASLD), and intended for use by physicians and other health professionals [89], do not recommend routine screening for NAFLD in high-risk groups (i.e., T2D or obesity) due to the uncertain evidence supporting diagnostic tests, treatment options, and the long-term benefits or cost-effectiveness of screening. Some specialists in the United States recommend screening individuals at risk of developing liver disease, such as those older than 50 years and with T2D or metabolic syndrome, using liver function tests and abdominal ultrasound in a primary care setting and imaging or prediction algorithms to assess the presence of fibrosis and subsequent diagnosis of NASH and staging of fibrosis [132].

**Table 3:** Summary of AASLD practice guidelines for the screening, evaluation, and treatment of NAFLD [89]

Screening	Routine Screening for NAFLD in high-risk groups attending primary care, diabetes, or obesity clinics is not advised because of uncertainties surrounding diagnostic tests and treatment options, along with lack of knowledge related to long-term benefits and cost-effectiveness of screening
	There should be a high index of suspicion for NAFLD and NASH in patients with type 2 diabetes
	Systematic screening of family members for NAFLD is not recommended
Evaluation	Patients with unsuspected hepatic steatosis detected on imaging who have symptoms or signs attributable to liver disease or have abnormal liver chemistries should be evaluated as though they have suspected NAFLD and worked up accordingly
	Patients with incidental hepatic steatosis detected on imaging who lack any liver-related symptoms or signs and have normal liver biochemistries should be assessed for metabolic risk factors (e.g., obesity, diabetes mellitus, or dyslipidemia) and alternate causes for hepatic steatosis, such as significant alcohol consumption or medications
	When evaluating a patient with suspected NAFLD, it is essential to exclude competing etiologies for steatosis and coexisting common chronic liver disease
	In patients with suspected NAFLD, persistently high serum ferritin, and increased iron saturation, especially in the context of homozygote or heterozygote C282Y HFE mutation, a liver biopsy should be considered
	High serum titers of autoantibodies in association with other features suggestive of autoimmune liver disease (> 5 upper limit of normal aminotransferases, high globulins, or high total protein to albumin ratio) should prompt a work-up for autoimmune liver disease
	Initial evaluation of patients with suspected NAFLD should carefully consider the presence of commonly associated comorbidities such as obesity, dyslipidemia, insulin resistance or diabetes, hypothyroidism, polycystic ovary syndrome, and sleep apnea
Treatment	Weight loss generally reduces hepatic steatosis, achieved either by hypocaloric diet alone or in conjunction with increased physical activity. A combination of a hypocaloric diet (daily reduction by 500–1000 kcal) and moderate-intensity exercise is likely to provide the best likelihood of sustaining weight loss over time
	Weight loss of at least 3–5% of body weight appears necessary to improve steatosis, but a greater weight loss (7–10%) is needed to improve the majority of the histopathological features of NASH, including fibrosis
	Pharmacological treatments, such as pioglitazone and Vitamin E, aimed primarily at improving liver disease should generally be limited to those with biopsy-proven NASH and fibrosis. Bariatric surgery can be considered in otherwise eligible obese individuals with NAFLD or NASH

A review of current international guidelines has recently been published [133]. In contrast to the AASLD, European and Asian guidelines recommend that screening be considered for groups considered at risk for developing NAFLD, including patients with obesity and T2D [3, 90, 91]. With respect to lean individuals, guidelines for NAFLD screening become less clear. Many guidelines acknowledge the importance of NAFLD in lean individuals, especially those of Asian ancestry or who exhibit features of metabolic syndrome [3, 90, 134]. The development and distribution of *consistent* screening and risk assessment guidelines will be critical to ensure optimal clinical management for all NAFLD patients [133]. Knowledge of disease etiology, screening, detection methods, and consensus guidelines are becoming increasingly important for adequate clinical care of both lean and obese NAFLD patients, especially for primary care physicians, who are the providers in the best position to make an initial diagnosis.

Most of the guidelines do not directly address screening and treatment of NAFLD in lean individuals. There are thus many questions that arise when considering the screening and clinical management of NAFLD in this patient population. For example, is visceral adiposity, rather than the overall amount of body fat, more relevant for NAFLD pathogenesis in lean individuals than it is in those with higher BMI? If so, are there better alternatives to the use of BMI as a marker for adiposity for NAFLD screening? Some investigators have argued that waist circumference is a more accurate representation of body fat distribution and a better method with which to identify individuals at higher risk of developing cardiometabolic disease [135]. However, despite the relative simplicity and low financial cost, implementation of waist circumference measurements as a standard measure of adiposity in primary care faces systemic obstacles, and in many settings, would require problematic process reconfiguration. However, obtaining waist circumference measurements may be clinically significant for lean individuals, who, despite having a normal BMI, may have some degree of visceral adiposity and consequently, an increased risk for NAFLD.

A major question is whether NAFLD in lean individuals represents a distinct clinicopathological entity requiring specific management, as suggested by many researchers [16, 27, 31, 32, 36, 136], or is it a sub-phenotype of classical obesity-associated NAFLD that will respond to the current approach of weight loss and control of insulin resistance, hypertension, and hyperlipidemia [17]? Certainly, many of the same factors that increase susceptibility to NAFLD are shared between normal weight and overweight individuals. Further, even among normal weight individuals, those with NAFLD appear to have slightly worse metabolic features. For example, Kim et al. [14] observed significant differences in sex, waist circumference, triglyceride level, and logarithm HOMA-IR between normal weight subjects with and without NAFLD. These differences in clinical and laboratory measures between normal weight individuals with or without NAFLD were comparable to those observed between overweight individuals with or without NAFLD, suggesting that in this cohort, NAFLD in lean individuals is a clinical entity similar to obesity-related NAFLD [14]. However, the risk of insulin resistance, hypertriglyceridemia, hyperuricemia, and central obesity in NAFLD patients compared with those without NAFLD was higher in lean individuals than those with overweight.

Lean individuals with NAFLD have also been found to respond to diet and lifestyle modifications typically utilized in the treatment of obesity-related NAFLD. In one study, loss of only 5% of initial body weight was demonstrated to result in remission of NAFLD in 75% of individuals [137]. Likewise, a 5% reduction in weight in 35 NAFLD patients (14 lean and 21 with obesity) yielded significant improvements in ALT and AST levels, hepatic steatosis, and liver stiffness [138]. In this intervention, NAFLD was resolved in 57.1% of lean individuals [138]. Combined, these data suggest that lifestyle modifications and weight loss are appropriate to reduce NAFLD, at least in some lean individuals. More research to determine whether reductions in central obesity, through a nutritional regimen and exercise, are appropriate therapeutic approaches in lean individuals with NAFLD.

It will also be important to determine whether therapeutics under investigation for classical obesity-related NAFLD will also be effective in lean NAFLD patients. Clinical trials addressing the potential effectiveness of drugs such as SGLT2 (sodium–glucose transport protein 2) inhibitors, GLP-1 (glucagon-like peptide 1) receptor agonists, obeticholic acid, pioglitazone, or saroglitazar in lean individuals with NAFLD are urgently needed [139].

Despite the similarities in NAFLD across the BMI spectrum, there may be cases in which NAFLD in lean individuals represents a distinct disease entity, and here, interventions that specifically address the pathophysiological triggers must be developed and tested. In these cases, the etiology and pathogenesis of NAFLD may inform the most appropriate treatment strategy. More studies to identify potential genetic factors that specifically contribute to NAFLD without obesity (or are masked by the presence of obesity) and uncover interactions with lifestyle factors that modulate their impact would provide a deeper understanding of disease risk in lean individuals. The role of various dietary factors or specific macronutrient composition as significant contributors to NAFLD risk in lean individuals remains largely unexplored. Many research studies have consistently demonstrated a link between liver dysfunction and choline deficiency, which has the unusual phenotype of resistance to diet-induced obesity, but are there other micronutrients that contribute to NAFLD, and if so, do they interact with functional genetic variants, as observed between choline and *PEMT* SNVs? Finally, there may be additional, as-yet unknown environmental factors, including herbal supplements, that contribute to the development of NAFLD in lean individuals.

Recent efforts to apply data-driven cluster analysis identified five distinct subtypes of diabetes, showing distinct patient characteristics and differential risk for diabetic complications [140]. This level of stratification of patients with a notably heterogeneous disease may lead to more focused treatment strategies instead of a one-size-fits-all approach, which represents the current state of diabetes care. We envision the application of similar cluster analysis to identify a spectrum of individuals from those who may have relatively mild NAFLD with little chance of progression to those who are on a rapid trajectory to advanced disease with severe complications. Such stratification may also lead to specific treatment strategies.

Going forward it will be important to assess the variance in NAFLD prevalence in lean populations, according to different ancestries, ethnicities, and geographies, and determine risk factors that may be more important for some groups than for others. Knowledge of the long-term consequences of NAFLD in the lean and the rate and severity of progression to NASH compared to classical obesity-associated NAFLD will also be important for the development of precise treatment strategies.

## Conclusions

It is not uncommon for lean individuals to develop NAFLD and NASH, despite a healthier metabolic phenotype than that observed in classical obesity-related NAFLD. We posit that NAFLD develops in lean individuals due to a distinct array of contributing etiologies, including dietary composition, genetic susceptibility, and hormonal status. In the absence of suspicious laboratory findings, lean individuals are not likely to be screened for NAFLD, nor for metabolic diseases associated with NAFLD. Awareness of menopausal status, genetic factors, ethnicity, dietary factors (especially added sugar, refined carbohydrates, and saturated fat/cholesterol), choline deficiency, and alcohol consumption patterns may be of value in assessing NAFLD risk in lean individuals.

Much more work is needed not only to address the questions raised above, but also to promote greater awareness among practitioners about the potential health risks associated with NAFLD in lean individuals. Efforts aimed at the development of screening algorithms that are less dependent on BMI and hepatic transaminase levels, implementation of more precise treatment strategies based on underlying pathoetiology, and inclusion of lean individuals in NAFLD-related clinical trials are necessary to reduce the burden of NAFLD in this patient group. Further, additional studies to characterize the lean NAFLD population and identify factors that modulate NAFLD risk in the absence of clinically significant metabolic dysfunction are urgently needed. Finally, recognition that NAFLD in some lean individuals may resemble classical obesity-related NAFLD, while in others, it may represent a distinct clinical entity, provides a foundation by which different strategies for clinical management can be devised. Early detection, combined with the appropriate steps to mitigate NAFLD through lifestyle modifications and clinical interventions, may effectively prevent the progression to NASH in lean individuals.

## Data Availability

Not applicable.

## Abbreviations

NAFLD: Nonalcoholic fatty liver disease
BMI: Body mass index
NASH: Nonalcoholic steatohepatitis
CI: Confidence interval
HDL-C: High-density lipoprotein-cholesterol
HOMA-IR: Homeostatic Model Assessment for Insulin Resistance
NHANES III: National Health and Nutrition Examination Survey III
LDL-C: Low-density lipoprotein-cholesterol
HCC: Hepatocellular carcinoma
ALT: Alanine aminotransferase
AST: Aspartate aminotransferase
GGT: Gamma-glutamyl transferase
AI: Adequate Intake
PEMT: Phosphatidylethanolamine *N*-methyltransferase
VLDL: Very low-density lipoprotein-cholesterol
SNV: Single nucleotide variant
PNPLA3: Patatin-like phospholipase domain-containing protein 3 gene
MBOAT7: Membrane-bound *O*-acyltransferase domain-containing 7
*TM6SF2*: Trans-membrane 6 superfamily antigen 2
OR: Odds ratio
AASLD: Association for the Study of Liver Diseases
SGLT2: Sodium–glucose transport protein 2
GLP-1: Glucagon-like peptide 1
